# Observations on Collodion in the Treatment of Diseases of the Skin

**Published:** 1849-02

**Authors:** 


*Observations on Collodion in the Treatment of Diseases of the Skin.* By Erasmus Wilson, Esq., F. R. S.

It is now about four months since a solution of gun-cotton in sulphuric ether (collodion) was placed in my hands by Messrs. Bell, of Oxford street, and since I first proceeded to employ it in the treatment of cutaneous diseases. I was at that time much interested in the medical progress of a young lady (the daughter of a physician in the west of England) who had been suffering for many years with scrofulous ulceration of the skin in various parts of the body. She had been under my care for several months, and the sores were much improved, but they were nevertheless very far from being healed. The diseased skin had the appearance of being worm-eaten, its hollows were filled with pus, which burrowed beneath the surface, and it was moreover thickened and congested. By the constitutional treatment which I had pursued, I had to a considerable degree corrected the pyogenic tendency of her system; but I felt the want of a local remedy that would serve as an impermeable covering to the surface—in fact, take the place of the lost epidermis, and act the part of an artificial scarf-skin. I had tried vulcanized caoutchouc spread with adhesive plaster, gutta percha, nitrate of silver, astringent solutions, ointments, and pressure by bandage, in vain—the remedy was not as yet found. I was revolving this in my mind when the collodion was put into my hand. The bearer of the little bottle may remember my exclamation, that "that was exactly the thing I wanted."

On the next visit of my patient, I removed the dressings from the sores, and pencilled them over with the new agent, which covered the surface with a powerfully adhesive film, of about the thickness of gold-beater's skin, and effectually represented the lost scarf-skin. A piece of dry, soft linen was the only additional covering required, and she left me, much delighted at the abandonment of the local applications and bandages. This young lady has since continued to apply the collodion herself, night and morning, until the present time, when the sores are nearly well, and the congestion and scrofulous thickening of the skin almost gone.

From careful observation of the effects of the collodion in this case, I found it to possess four important properties, namely:—

First. That of a mild stimulant.

Second. That of an efficient substitute for the natural scarf-skin.

Third. That of a mechanical compress.

Fourth. That of an adhesive glue, from which quality it derives its name.

First. As a mild stimulant, it is fitted to exert a local alterative action on the congested capillaries of a chronic ulceration, and give activity to the healing process.

Second. In its character of a substitute for the absent scarf-skin, it is transparent, pliant, and more or less impermeable, according to the thickness of the layer that may seem to be required.

Third. Its most remarkable property, as it seems to me, is the contraction which occurs during the desiccation of the collodion, and which produces a local pressure of considerable power on the surface to which it is applied. Thus, in the case above related, the congestion of the thickened skin was relieved by the varnish-like film of collodion spread upon its surface, by means of a camel-hair brush, as completely as if a nicely-adjusted bandage had been placed over it. In another instance, I found a film of collodion entirely remove a purple congestion (resulting from imperfect circulation) from the tip of the nose, in a lady who had long suffered from the annoyance. In a third case, in which the fingers of an elderly lady were congested and blue, and the congestion was attended by pain and throbbing, like that which accompanies chilblains, the collodion produced so much contraction as to render their tips white and bloodless, and I was obliged to discontinue the application in consequence.

Fourth. The glue-like property of the collodion is evinced in its adhesion of cut surfaces, a property which is much increased by the contraction above mentioned. When employed with the purpose of keeping together the edges of an incision, a piece of cambric or thin linen rag should be dipped in the solution, and placed along the line of incision, after the cut edges have been adjusted and carefully dried, perfect dryness of skin being a necessary condition to the adhesion of the solution. From the rapidity with which the solution dries, and its perfect adhesive powers, collodion is likely to occupy an important place among the "adjuvantia" of surgical practice.

The diseases of the skin in which I have hitherto used the collodion with advantage are, chronic erythema of the face; intertrigo; chapped nipples and chapped hands; herpes labialis, preputialis and herpes zoster; lichen agrius; lupus non exedens and exedens; acne vulgaris; and several affections of the sebiparous organs. In chronic erythema of the face, its contracting power was most usefully evinced, as it was also in lupus non exedens and acne.

In a troublesome case of chapped hands and fingers, resulting from chronic lichen agrius, the collodion acted not merely as a protective covering, but also promoted the healing of the cracks more quickly than the remedies I have been in the habit of employing. In chapped nipples, it was even more efficient in its protective and curative action, and seemed, in the two instances in which I used it, to work a charm upon the painful skin. The gaping cracks were instantly drawn together, and almost obliterated by the contracting power of the remedy, and were effectually shielded from the influence of moisture, and the pressure of the gums of the infant, and all this in consequence of the rapid evaporation of the ether, in an instant of time. In another point of view the remedy is invaluable as an application to chapped nipples, namely, as being in nowise injurious to the infant, from offering nothing which can be removed by the lips during the act of sucking, and in this particular, therefore, possessing a vast superiority over the various forms of ointments, astringent lotions, &c.

In four instances it immediately put a stop to herpes labialis, and in a very severe attack, it showed itself to be a powerful and useful remedy. Small superficial ulcerations of the corona glandis and prepuce, caused by excoriation, were cured by a single application, and in a gentleman very susceptible of excoriation, it acted admirably as a prophylactic. From the success of the latter trial, I am inclined to think that it might be usefully employed as a prophylactic, in cases of exposure to syphilitic contagion.

When properly applied, the collodion enters all the crevices of the lines of motion of the skin, and adheres so firmly as to require several washings for its removal. As it is usually prepared, it has the consistence of syrup, and in this state is best suited for those cases in which its adhesive properties are principally needed. Where, however, it is intended to be applied to the surface of an ulcer or abrasion, or to chaps of the skin, I find it convenient to dilute it with ether, and render it almost as limpid as water.

In pursuing this subject, I have made trial of a solution of gutta percha in chloroform, and also in benzone, but these solutions are very inferior to the collodion, for the purposes above named. Their adhesive powers are weaker than the collodion, and the layer which they form when painted on the skin, is apt to rise at the edges and rub off.—*London Lancet.*

